# Failing to get the gist of what's being said: background noise impairs higher-order cognitive processing

**DOI:** 10.3389/fpsyg.2015.00548

**Published:** 2015-05-21

**Authors:** John E. Marsh, Robert Ljung, Anatole Nöstl, Emma Threadgold, Tom A. Campbell

**Affiliations:** ^1^Department of Building, Energy, and Environmental Engineering, Faculty of Engineering and Sustainable Development, University of GävleGävle, Sweden; ^2^School of Psychology, University of Central LancashirePreston, Lancashire, UK; ^3^Psychology, City UniversityLondon, UK; ^4^Neuroscience Center, University of HelsinkiHelsinki, Finland

**Keywords:** noise, elaborative processing, false recall, semantic clustering, speech intelligibility

## Abstract

A dynamic interplay is known to exist between auditory processing and human cognition. For example, prior investigations of speech-in-noise have revealed there is more to learning than just listening: Even if all words within a spoken list are correctly heard in noise, later memory for those words is typically impoverished. These investigations supported a view that there is a “gap” between the intelligibility of speech and memory for that speech. Here, the notion was that this gap between speech intelligibility and memorability is a function of the extent to which the spoken message seizes limited immediate memory resources (e.g., Kjellberg et al., 2008). Accordingly, the more difficult the processing of the spoken message, the less resources are available for elaboration, storage, and recall of that spoken material. However, it was not previously known how increasing that difficulty affected the memory processing of semantically rich spoken material. This investigation showed that noise impairs higher levels of cognitive analysis. A variant of the Deese-Roediger-McDermott procedure that encourages semantic elaborative processes was deployed. On each trial, participants listened to a 36-item list comprising 12 words blocked by each of 3 different themes. Each of those 12 words (e.g., bed, tired, snore…) was associated with a “critical” lure theme word that was not presented (e.g., *sleep*). Word lists were either presented without noise or at a signal-to-noise ratio of 5 decibels upon an A-weighting. Noise reduced false recall of the critical words, and decreased the semantic clustering of recall. Theoretical and practical implications are discussed.

## Introduction

In everyday life, listeners have to recognize speech under conditions in which the speech signal is degraded, masked or even replaced by the presence of background sound. From traffic in the street to cross-talk in a restaurant, that unwanted background sound is termed “noise”. The impact of such noise on hearing aid users is socially profound. The overwhelming majority of patients visiting a hearing healthcare professional have reported difficulty understanding conversation in noise (Kochkin, 2000). Indeed, a large-scale survey revealed that one quarter of consumers did not use their hearing aid because of noise (Taylor, 2003). Adaptive procedures measuring performance in response to noise-embedded sentences are progressively becoming understood as essential to the routine diagnostic battery throughout the hearing aid fitting and selection process (Nilsson et al., 1994; Taylor, 2003; Killion et al., 2004).

For individuals with sensorineural hearing impairment, in turn with degraded input to their auditory systems, speech intelligibility may be affected by peripheral energetic masking (Kidd et al., 1998), further degrading the information in that signal even before neural transduction of that information at the cochlea. That neuronally transduced input may be regarded as even more partial because of the peripheral presence of noise. To a certain extent, the brain has been shown to use context to repair degraded sensory information and thereby improve speech perception (Shahin and Miller, 2009; Shahin et al., 2012). Further, speech-in-noise training has been shown to improve the performance on speech-in-noise tasks containing sentences (Song et al., 2012). Cognitive factors enhancing the cues that are important to listening to speech-in-noise caused this improvement, as was measured at the level of responses of the auditory brainstem (Skoe and Kraus, 2010; Campbell et al., 2012). Such performance improvements occurred with speech-in-noise training of cochlea implant users, who receive the very partial input of vocoded speech to a few electrodes at the cochlea (Ingvalson et al., 2013). That is, this speech-in-noise training caused improvements of central origins. The current research is thus centrally focused. A form of central masking of speech intelligibility (e.g., Kidd et al., 2010) affects the perception of individual words of sentences. At this level, central masking does not degrade the signal but rather a central interference occurred between the noise and the to-be-attended signal. Crucial to the brain repairing a degraded speech signal is top-down prediction. That prediction could be influenced by phonemic, syntactic, and semantic information.

Germane to this concept of top-down prediction has been evidence that syntactic complexity raises speech reception thresholds in fluctuating noise in a manner less apparent with stationary noise (Uslar et al., 2013). A kindred cognitive phenomenon has been observed through examining the impact of noise effects on working memory performance (Schlittmeier et al., 2012) whereby noise that fluctuates more, proved more disruptive. Accordingly, fluctuating noise disrupted the cognitive mechanisms involved in retaining the memory for words, in turn, disrupting performance on Uslar et al.'s (2013) speech-in-noise task. Such noise disrupts visually based tasks even when semantically unrelated to the task being performed and even when heard at a low-to-moderate intensity (Marsh and Jones, 2010; for reviews, see Hughes and Jones, 2001, 2003; Beaman, 2005; Campbell, 2005; Beaman et al., 2007; Szalma and Hancock, 2011). With the advent of overly populated schools and open-plan offices, concern is rising that increases in noise pollution are adversely affecting scholastic attainment (Klatte et al., 2013) and productivity at work (Mak and Lui, 2012). What is really needed to understand distraction is an account of the effects of noise on perceptual processing (e.g., hearing), cognitive (mnemonic) functioning and the interplay between the two. With the emergence of the field of cognitive hearing science, research has identified that the capacity for language understanding is affected both by, on the one hand, processes that are perceptual and bottom-up, and, on the other hand, processes that are cognitive and top-down (e.g., Rönnberg et al., 2008). Pivotal to this cognitive hearing science approach is how changes in speech understanding (e.g., intelligibility) are underpinned by perceptual and cognitive functions.

A recent investigation has brought auditory noise to the fore in cognitive hearing science. This investigation concerned the effects of noise on the perception of words and the subsequent memory for those words (Kjellberg et al., 2008). Participants, who heard words correctly within noise, recalled those words worse than when those words had been presented without noise. That is, noise, which did not impair identification of speech, impaired cognition. The current study investigated how listening to spoken words in noise takes working memory resources away from the encoding, storage, and further processing of those words (Kjellberg et al., 2008). A variant of the Deese-Roediger-McDermott paradigm (DRM; Deese, 1959; Roediger and IIIMcDermott, 1995) was employed. Within the memory and language literature DRM procedures have been used to measure semantic processing (Stadler et al., 1999; Johansson and Stenberg, 2002). The present experiment gauged whether listening in noise reduced such semantic processing despite accurate identification of each spoken word during study.

Working memory deficits amongst the elderly have been attributed to degraded linguistic input due to age-related hearing loss (Rabbitt, 1968, 1991; Cervera et al., 2009). Speech understanding in effortful listening conditions, either due to background noise, or age-related hearing loss, is considered to require the direction of processing resources toward perceptual processing. That processing is required for recognizing the speech material. As a consequence even if the recognition of speech is successful, fewer resources are left to accomplish other tasks such as storage, manipulation (e.g., elaboration), and comprehension of the materials. This “effortful listening” hypothesis is supported by the fact that adding broadband background sound (e.g., white noise) to a list of to-be-remembered spoken words—thereby reducing the signal to noise ratio or SNR—can impair free recall. That is, even when those words have been correctly heard previously, such noise still produced an impairment of memory performance (Kjellberg et al., 2008; Ljung and Kjellberg, 2009; Ljung et al., 2013; see also Rabbitt, 1968; Pichora-Fuller et al., 1995; Murphy et al., 2000; Schneider et al., 2002; McCoy et al., 2005; Wingfield et al., 2005). Since these noise effects cannot be attributed to impaired identification of material at study, it has been proposed that noise makes word identification more difficult, thus leaving fewer working memory resources available for the encoding, storage, and further processing of the words (McCoy et al., 2005; Kjellberg et al., 2008). Very few studies have investigated whether listening in noise impairs semantic processing of spoken words despite the correct identification of those words in noise. The paucity of research in this area impelled us to address the claim that listening to spoken words in noise reduces the higher-order cognitive processing (e.g., semantic processing) of those words. Here, we used a free recall task in which we presented lists of thematically-related words (e.g., Stadler et al., 1999; Johansson and Stenberg, 2002). This variant of the DRM procedure is known to elicit higher-order (e.g., gist-based, or relational) semantic processing. If noise were to reduce such processing, noise would affect aspects of free recall performance as captured by this DRM procedure.

In the DRM procedure, participants are presented with a list of items (e.g., *bed, tired, snore…*) that are all associated with a critical, non-presented, word, or theme (e.g., *sleep*). Many studies show that participants tend to falsely recall this critical lure despite explicit instruction not to guess (Deese, 1959; Roediger and IIIMcDermott, 1995). According to one approach (e.g., Brainerd et al., 2008), these “associative illusions” constitute a reflection of semantic gist processing: the semantic gist of the list is used as a retrieval cue (e.g., that all the words in the list were examples of “fruit”) and the critical word is “recalled” because it matches that cue. Gist-based processes are distinguished from verbatim processes, the latter of which are responsible for encoding surface details (e.g., that the word was “banana”, that the word was presented in black, printed in lowercase, etc.). Similarly, relational processing—thinking about the commonalities of list words—also increased the false recall of the critical word (e.g., McCabe et al., 2004). According to Kjellberg et al. (2008) and McCoy et al. (2005), noise could have stinted such gist-based processing that involves deep-encoding processes. If this view is correct, then noise is predicted to reduce the frequency of false memories.

Higher cognitive processes are not only marked by the occurrence of false recall, but also by the organization of responses on that free recall task. In the typical DRM procedure approximately 15 words are associated with a critical word item (or “theme”; e.g., *sleep*). These 15 words are presented for free recall, but the critical item is not presented. Semantic processing is therefore indexed by this DRM procedure and this processing is revealed by the apparition of the lure critical item as a response. To further reveal the semantic organization of the list words during output, we modified the DRM procedure such that groups of words were associated to one of three different themes within the same list (e.g., consider a number of words: *dream*, *bed*, *wake*, top, peak, summit, **hate**, **fear**, **cross**, presented from three themes: *sleep*, mountain, **angry**). For free recall, participants are expected to spontaneously cluster list words by theme at a greater-than-chance level even in the absence of explicit instruction to cluster. Further, participants typically cluster their responses by theme or by category even if, during study, the words are randomized with respect to their theme or categories (Bousfield, 1953; Smith et al., 1981; Marsh et al., 2009, 2014). The advantage of using this procedure, over free recall of lists comprising associates to a critical lure, is that this modified DRM yields a measure of semantic processing at test. That is, the degree of organization of responses by semantic category serves as an index of semantic processing (e.g., Marsh et al., 2014). This semantic-clustering provides an opportunity to assess the degree to which extant semantic associations guide the encoding and retrieval of episodic information (Bousfield, 1953; Tulving, 1968). Semantic-clustering is typically enhanced when processing is directed toward the organizational relations among list items as a whole. That is, if participants attempt to concentrate on what the words within the study list have in common with one another—relational processing (arguably the default strategy when to-be-remembered words from the same theme are grouped together [blocked by theme] during study)—semantic clustering is enhanced (Hunt and Einstein, 1981; Hunt and McDaniel, 1993). It is also known that such blocking of words by thematic category gives rise to a greater number of false memories (e.g., Goodwin et al., 2001; McCabe et al., 2004).

Investigations have previously revealed side effects of poor listening conditions on mnemonic retention (e.g., Kjellberg et al., 2008; Ljung et al., 2009). The novelty of the present investigation resided in testing whether poor listening impairs higher-order semantic processing (gist-based processing or relational processing) of to-be-remembered spoken material. Prior studies have manipulated the SNR of spoken material. For example, in the speech reception threshold test (Plomp and Mimpen, 1979) participants attempt to recognize and repeat familiar words (e.g., baseball, playground) or sentences presented at decreasing intensity typically against a background of noise; low SNRs occur when the difference between signal and noise decreases. At such low SNRs the listener has to rely more on informational redundancy and contextual cues to understand the word or sentence. A typical approach to determining the speech reception threshold has been to ascertain the level at which the participant can accurately repeat 50% of the presented words. Kjellberg et al. (2008), however, used a variant of this procedure to investigate the listening-memory function for words heard in noise. Kjellberg et al. drew two phonetically balanced word lists of 50 monosyllabic words from standardized audiometric materials (Hagerman, 1982). Words within each list were semantically unrelated to one another, so as to minimize the contribution of semantic and contextual top-down cues to listening. In the noise condition, aperiodic noise accompanied the list of 50 spoken words (SNR = 5). The rate of presentation was one spoken word every 4 s. Participants attempted to repeat back each word after that word was presented to ensure correct identification. The participants were aware of the requirement to memorize the words for a later memory test. Recall was immediate, following directly after the presentation of the list. Kjellberg et al. showed that adding noise to the spoken words impaired their free recall despite the fact that each word had been repeated earlier to ensure correct identification. This empirical acoustic setting, according to the ease of language understanding model (ELU; Rönnberg et al., 2008) rendered insufficient the implicit and unconscious cognitive-linguistic processing of the spoken words. The processing did not suffice to support the identification and understanding of those words. As a consequence, explicit processes requiring working memory are required to match degraded input to long-term memory representations by inferring missing information or repairing misunderstandings. Accordingly, that repair occurred either in a prospective manner, predicting what is upcoming within spoken language, or retrospective manner identifying what has already been said. These explicit working memory processes were accordingly cognitively demanding and necessary for the correct identification and comprehension of the speech signal under such adverse conditions (Rönnberg et al., 2009). Associated with these cognitive demands is a reduced availability of episodic memory resources. Such functions would have otherwise supported concurrent or subsequent storage, alongside the elaboration (e.g., relational or gist-based processing) of the speech input. Hence, later memory for the words suffered. In this conceptualization, therefore, speech perception consumed mnemonic functions—particularly if that speech is degraded or one has hearing difficulty. Hence individual differences in memory functions have a pronounced effect on perceptual processing and reception of speech. Evidence supporting such an approach has stemmed from noise disrupting memory in the context of recognition, paired associate learning, sequential recall of nonsense syllables, sentence recall, discourse comprehension, and comprehension of oral instructions (Rabbitt, 1968; Pichora-Fuller et al., 1995; Surprenant and Neath, 1996; Murphy et al., 2000; Surprenant, 2007; Klatte et al., 2010; Valente et al., 2012). In all such investigations, a memory disruption occurred even though the SNR allowed perfect, or near perfect, identification of the speech. The individual susceptibility of memory functions to interference by noise thus determined how prior context can be used to predict and, in turn, repair and retroactively interpret speech in noise.

The purpose of this investigation was thus to test the hypothesis that listening to spoken words in noise reduces the semantic processing of those words. This we term the *effortful listening hypothesis*. More specifically, it is postulated that noise disrupts working memory, in turn, affecting semantic processing.

## Experiment

The current investigation thus developed the paradigm of Kjellberg et al. Rather than using short (e.g., 10-item) lists of unrelated words that would not permit analyses of semantic processing, a relatively long list of words was employed. Each list of words was blocked according to three themes. Prior studies demonstrate that blocking lists by semantic associates promotes spontaneous processing of the semantic relations between items within a list (e.g., D'Agostino, 1969). The aims of using blocking, here, was to increase the likelihood that participants would organize responses by category and to precipitate false memories (e.g., McCabe et al., 2004). With such lists containing several themes, participants, at test, are known to cluster the associatively-related items together at a greater-than-chance level even in the absence of any instruction to do so (e.g., Bousfield, 1953). Both false recall and semantic clustering are thus expected to yield evidence that participants bring to bear pre-existing conceptual relationships or semantic associations to guide encoding and retrieval of episodic information. If, according to the effortful listening hypothesis, noise renders word identification difficult—thereby leaving fewer working memory resources for the further processing and semantic elaboration of the words (e.g., McCoy et al., 2005; Kjellberg et al., 2008)—then it would be predicted that presenting lists of semantic associates in noise as compared to quiet (no noise) will reduce false memories. This effortful listening hypothesis also predicted that noise will reduce the degree to which the associates are thematically organized (clustered) at test.

### Method

The study was approved by the Regional Ethical Review Board at the University of Uppsala (Dnr 2011/108). As the data would be treated anonymously, and no apparent ethical research complication with participation could be identified, oral consent was deemed sufficient by the Ethical Review Board. The data collector took note of the oral consent of participants.

#### Participants

Thirty-one participants (18 male, 13 female) with a mean age of 26.8 years (range = 20–42 years) from the University of Gävle took part in return for a cinema ticket. Each participant self-reported normal hearing and Swedish as their first language. Data from five participants were excluded due to equipment failure (3 participants) or the occasional non-compliance with experimental instructions (2 participants).

#### Apparatus and materials

#### To-be-remembered material

Twelve associates were chosen from each of 30 themes in the Johansson and Stenberg (2002) norms in order to construct 10 lists of 36 words, each having 3 themes (see Appendix A in Supplementary Material). Themes chosen had minimal word overlap such as to diminish the possibility of proactive interference (Shuell, 1968). The words chosen were the 12 most frequently produced instances to the non-presented, critical item.

Themes were randomly assigned to each list. However, this assignment was with the constraint that associated themes were not presented together. Words within each list were arranged in a blocked format such that all associates from a given theme were presented together within the list. The words were digitally recorded without intonation in a female voice at an approximately even-pitch and sampled with a 16-bit resolution at a sampling rate of 44.1 kHz using Audacity software. The first and second author listened to the recordings to ensure that they achieved this criterion and for the few occasions in which the recordings of the spoken words failed to meet these criteria, the words were recorded again. Spoken items were presented at an equivalent sound level of 64 decibels as measured with a digital sound level meter (Mastech MS6700) on an A-weighting.

#### Noise

Broadband noise was synthesized from the speech material thereby producing noise with the same long-term-average-spectrum characteristics (LTAS) as the speech stimuli. For the noise condition, the noise at 59 decibels was mixed with the spoken items, thereby giving an SNR of 5 decibels. This SNR made listening demanding, but not impossible. The lists were presented via stereo headphones that the participants wore throughout the experiment. Participants wore headphones throughout the control condition and the background noise within the room yielded an SNR of 28 decibels. Measures in decibels were determined using an A-weighting.

#### Design

A repeated-measures design was used with one within-participant factor Noise, of which there were two levels: No noise, Noise. The two conditions were randomized as follows. The 10 lists were randomly split into two sets of five and interleaved with one another during presentation. For half of the participants, the first list was presented in noise and the second list presented in quiet (no noise) with trials alternating thereafter between noise and no noise, whereas this order was reversed for the other half of the participants.

#### Procedure

Participants were seated in a cubicle. Lists of theme items were presented over stereo headphones (Sennheiser HD-200) one word at a time with an inter-stimulus interval of 4 s of quiet between each word. Retrieval was immediate at the end of the list and participants typed their answers into an E-Prime computer program that also controlled stimulus presentation.

Participants were tested individually in a small room comprising a HP Compaq 6720s laptop PC. They were informed that they would be presented with ten 36-word lists and that each list would be presented one-word-at-a-time over headphones, from which they were asked to memorize as many words as possible and write the words they remembered down in the order that they came to mind. Participants were not explicitly told that the lists could be categorized by theme. Participants were informed that they had unlimited time for recall, and that when they could not remember any additional items, they should click on a “continue” button to initiate the onset of the next list. Participants were instructed that the to-be-remembered items would sometimes be accompanied by noise. They were also were instructed to ignore the noise and to concentrate on identifying each word. The experimental session lasted for approximately 50 min^1^.

#### Recall measures

Recall measures came in four forms: the total number of items correctly recalled, the mean number of items recalled by theme, the number of themes recalled (scored by recalling one word from a theme), and the number of critical items falsely recalled.

## Results

Table 1 shows the results of the various recall measures in the two conditions. The effects of noise on each dependent variable displayed were those predicted by the effortful listening hypothesis that effortful listening in noise detracts from the elaborate, semantic processing of spoken words. Accordingly, in noise, fewer items were recalled correctly, with fewer correct items per semantic theme and fewer such themes. Fewer critical lure words, not present in the list, were recalled. This further evidence for a noise-induced decrement in semantic processing was bolstered by the clustering measure detailed in the ensuing sub-section.

**Table 1:** **Mean recall performance for the four recall measures as a function of two background conditions (no noise vs. noise) used in the study**.

**Dependent measure**	**No noise**		**Noise**	
	***M***	***SD***	***M***	***SD***
Mean number of spoken words correctly recalled per list	10.45	0.67	8.78	0.58
Mean number of spoken words per theme correctly recalled per list	4.41	1.09	3.60	0.89
Mean number of themes correctly recalled per list	2.62	0.34	2.48	0.42
Total number of critical lures recalled	1.46	0.19	2.42	0.37
Thematic (Semantic)-clustering (*Z* scores)	2.43	0.20	1.95	0.16

Inferential statistical analyses corroborated these overall tendencies: the mean scores for the total number of items correctly recalled per list was significantly lower in the noise condition than in the control condition, *t*_(25)_ = 4.24, *p* < 0.001, *CI*_0.95_ = [0.858, 2.48]. Further, the mean number of items per theme recalled was also significantly lower in the noise condition than in the control condition, *t*_(25)_ = 3.52, *p* = 0.002, *CI*_0.95_ = [0.227, 0.868]. In addition, the mean number of themes recalled was also significantly lower in the noise condition than in the control condition, *t*_(25)_ = 2.47, *p* = 0.021, *CI*_0.95_ = [0.024, 0.268]. Finally, the mean number of critical lures recalled was also smaller in the noise condition as compared to the control condition, *t*_(25)_ = 2.83, *p* = 0.009, *CI*_0.95_ = [0.262, 1.66]. There were too few intrusions to be subject to inferential statistical analysis. This paucity of such intrusions is likely due to the theme of the list acting as a top-down guide such that words phonemically similar to targets were not produced because those words did not “fit” with the semantic theme being recalled.

### Clustering measure

There are a number of ways of measuring semantic clustering (Murphy, 1979). Here, we use the *Z* score (for the mathematical assumptions and algorithms used to compute *Z* scores, see Frankel and Cole, 1971). Briefly, this measure of clustering is based on the number of runs of exemplars of the same category at test. Run length is defined as the number of same-category items recalled in succession. Items recalled in isolation are scored as runs of one. Therefore, the number of runs is one more than the number of times the category changes during recall. Suppose *a*, *b*, and *c*, represent different themes and items from the themes: The recalled list *aaabbccbcbb* has six runs commencing with a run of three and terminating with a run of two. On the *Z* score measure, clustering occurs when the number of runs that are observed on the output list is significantly fewer than expected by chance. Perfect clustering, e.g., *aaaabbbbcccc*, results in a higher *Z* score than imperfect clustering, e.g., *abaabccbbcca*. Positive scores indicate tendencies toward clustering. Negative *Z* scores are possible when less categorization occurs than by chance. The *Z* score, therefore, has an advantage over several other methods of assessing clustering because the *Z* score enables one to tell if clustering is at an above-chance level (Frankel and Cole, 1971). *Z* scores here, as is typical (Murphy, 1979), were computed with all repeat and intrusion errors removed. The mean *Z* score was lower in the noise condition than in the no noise control condition, *t*_(25)_ = 3.19, *p* = 0.004, *CI*_0.95_ = [0.169, 0.788].

## Discussion

The results show that the effortful listening hypothesis is supported. That is, listening in noise is effortful and requires working memory resources that are necessary for elaborate, semantic processing of spoken words (McCoy et al., 2005; Kjellberg et al., 2008). Noise disrupts that elaborate semantic processing. With speech-in-noise, participants not only remember less of the word lists, but also falsely recall fewer critical items, fewer themes, and semantically cluster less of the associates by theme at output when the words were presented in noise. The recall of critical items, themes, and semantic clustering is traditionally accepted as reflecting higher-order semantic processing (Hunt and McDaniel, 1993; Burns and Brown, 2000). The reduction in recall of themes, for example, is thought to reflect the failure to adequately establish higher-order semantic encodings during study that can be used during retrieval as a plan to enable the transition between themes during recall (Bower et al., 1969). Listening difficulty thus requires working memory resources thereby leaving fewer of these limited resources available for encoding, storage, and further conceptual processing of the words using pre-existing semantic associations (McCoy et al., 2005; Kjellberg et al., 2008).

Consistent with the ELU model (Rönnberg et al., 2008), the interpretation offered is that listening in noise renders the implicit lexical access processes that ordinarily underpin language processing insufficient. Therefore explicit processes requiring working memory resources are required to match, via reconstruction, the degraded incoming stimuli against representations in long-term episodic memory. These processes are guided by top-down knowledge that the list words belong to semantic themes, therefore avoiding the incorrect production of a candidate item that is phonologically similar to the target. However, this resource-demanding process adversely affects other resource-requiring processes involving episodic long-term memory. There is thus a compromise in the operation of that resource-requiring relational processing or gist-based processing (Serra and Nairne, 1993; DeLosh and McDaniel, 1996). That compromise has knock-on effects impairing storage and elaboration of the spoken input. Consistent with this view, one possibility is that listening difficulty during study increases the use of *verbatim processing* at the expense of *gist-based processing*. Noise, for example, may require participants to process the verbatim, perceptual characteristics or surface forms of the spoken words to identify those words. Such characteristics include information about the phonetic constituents of those words or the linguistic style of the speaker. This increase in verbatim processing thus leads to an impoverished gist-based processing of how the words belong to a common semantic theme. In turn, there is a reduction in the elicitation of false recall (cf., Brainerd and Reyna, 2002).

Another possible explanation for the impairment to recall that listening in noise produces may be found in relation to the distinctiveness processing framework (Hunt et al., 2011). This framework incorporates two episodic processes: *Relational processing* that encodes similarity among a set of items, and *item-specific processing* that encodes information that is specific to individual items. In the current study, the listening difficulty that noise causes could produce a focus toward the item-specific properties of the to-be-remembered items at the expense of relational processing. A reduction in relational processing would have the consequence of reducing semantic organization. That reduction would, in turn, cause less clustering by theme (Hunt and McDaniel, 1993) and a reduction in false recall (Hunt et al., 2011). Moreover, another possibility is that listening effort merely tilts the balance between relational and item-specific processing toward item-specific processing. A similar view has been espoused by Arndt and Reder (2003) to explain their finding that presenting each list item in a perceptually distinct font, as compared to the same font, reduced false memory for the critical lures. Arndt and Reder (2003) suggested that the unique fonts caused processing of item-specific features of the visual items. Processing of those item-specific features therefore directed processing away from the relational information. As a consequence, Arndt and Reder argue, the probability of activating the critical items is therefore reduced. With regard to the current study, our findings are consistent with the view that noise directs processing to the item thereby reducing relational processing. Indeed, this is consistent with the notion that noise induces a process that explicitly matches the incoming stimuli with representations in episodic long-term memory (Rönnberg et al., 2008). However, as Arndt and Reder (2003) do, we suggest that the processing balance between item-specific and relational processing is merely tilted in favor of item-specific processing, rather than increasing it. This conclusion is shaped by the finding that listening in noise reduced correct recall in the current investigation. By contrast, for manipulations and orienting tasks that emphasize item-specific processing, correct recall is typically facilitated (Mulligan, 1999) or unchanged (Hunt, 2003).

The results do not support the view that noise degrades the sensory traces of stimuli making them more difficult to discriminate from one another (Surprenant and Neath, 1996; Surprenant, 2007). According to this view the occurrence of false recall should be greater in the noise condition: Participants no longer have access to the acoustic codes that could distinguish the study items from the non-presented critical items that lack such a code (Rummer et al., 2009).

The current investigation offers further evidence for the gap between listening and mnemonic performance: Our pilot study showed a 4.5% reduction of correct identification by noise. However, there was almost a threefold drop in mnemonic performance (16%), which is substantially greater than the 7.7% reported by Kjellberg et al. (2008). We attribute this drop in mnemonic performance to the semantic nature of the task used in the current study. In the investigation by Kjellberg et al. (2008) lists comprised words that were *unrelated* to one another. This lack of a meaningful association presumably required a greater reliance on perceptual as compared with semantic coding of the speech signal. In the listening-in-noise condition, explicit matching processes would be required, whereby perceptually similar alternative interpretations of the speech stimuli are considered as using stored information (Rönnberg et al., 2008). Within the current experiment, lists of thematic words were used and these items were blocked by theme. The semantic priming occurring between consecutive items constrains the search set within long-term memory and diminishes any gain that may arise from generating phonetically similar, candidate items within long-term memory. Moreover, there is a possibility that our method of blocking list items by theme during study could have underestimated the disruptive effect of noise on some measures of higher-order processing: Blocked presentation methods are known to give rise to greater false recall levels than when themes are randomly interspersed throughout a list, or are presented along with unrelated filler items (Goodwin et al., 2001). However, blocking items by theme (or category) compared to random presentation increases semantic clustering (D'Agostino, 1969). Therefore, it is possible that much more pronounced effects of noise during study arise for semantic clustering if the associates to each theme are randomly presented throughout the list whereby the semantic connections to the themes are more difficult to process.

Working memory processes play a role when individuals with hearing loss listen in noise (Akeroyd, 2008; Rönnberg et al., 2008; for a review, see Mattys et al., 2012). More work is needed that investigates the memory functions for the semantically rich materials used in the current study for individuals that differ in relation to working memory capacity and hearing. Semantic effects are predicted to be more pronounced for individuals with poorer speech perception capabilities in noise. Such individuals include those with hearing impairment (Rabbitt, 1991; Pichora-Fuller et al., 1995) or young children (Wightman and Kistler, 2005, whom typically require an SNR 5-7 decibels higher to achieve similar levels of identification of speech and nonspeech signals, see Werner, 2007). Similarly individuals with low working memory capacity should also experience a much greater disruptive effect due to listening in noise. Previous work has also shown that advancing age can be offset by cognitive capacity, indicating that listening *per se* is maintained among elderly individuals with high working memory capacity (Rönnberg, 2003). However, as we have described earlier, listening success and later mnemonic success are different functions. Therefore, there is a requirement to understand whether mnemonic performance is impaired disproportionately among younger and older adults with comparable listening ability. Further, while younger individuals, do benefit from semantic encoding instructions as compared with shallow encoding instructions, with particular relevance to our current study, elderly, elderly individuals do not. For example, older adults show less activity in regions of the brain that are associated with semantic processing than younger adults (Daselaar et al., 2003). Elderly individuals as compared to younger adults are accordingly not only disproportionately impaired in listening to semantically rich material (Pichora-Fuller, 2008), but also in their memory for such material. The effects demonstrated here should also be more pronounced when the masking sound is fluctuating noise rather than steady noise (Leibold and Neff, 2007; Uslar et al., 2013), which is arguably more ecologically valid particularly within the built environment setting.

By contrast to the present investigation, the recent findings of Uslar et al. (2013) investigate speech reception thresholds of sentences rather than single words in noise. These thresholds in fluctuating noise are strongly correlated with cognitive abilities. That is, an individual's attention or “conflict monitoring” (Stroop) and working memory (digit span, word span) ability correlated with speech perception in that noise in a manner not shown for speech without noise. Uslar et al.'s (2013) findings thus support the view that, in noise, cognition “kicks-in” during speech understanding (Rönnberg et al., 2010). However, Uslar et al.'s (2013) data show these individual differences in cognitive factors neither influence how deviations from a canonical word order, nor how increases in syntactic complexity, affect speech recognition thresholds in fluctuating noise.

A question outstanding for cognitive hearing science is what role working memory plays in speech perception in noise if working memory does not assist the syntactic processing of sequences of words in sentences? The data of the present investigation address this question. Working memory for the semantics of prior material affect the lexical access and the elaborative processing of speech-in-noise. Accordingly, semantic processing operates predictively to determine what is heard in a top-down manner, permitting the brain to repair semantically predictable utterances obscured by noise. It is posited in the theory offered that semantic repair during lexical access in noise requires working memory resources. Not only does this requirement affect the perception of speech-in-noise but also the understanding of that speech—the primary objective of listening to speech.

In the longest-term, a test sensitive to the identified influences of semantic processing of speech-in-noise might join the audiologist's diagnostic battery including established approaches using sentences in noise. Such an approach could, at least, give the patient a realistic assessment of the structure of their communication problems, and how well particular hearing-assistive devices and cognitive training programmes such as working memory training (Henshaw and Ferguson, 2013) might, or might not, help. Further development of valid diagnostic measures related to semantic processing of speech-in-noise is required. If those measures offer a specificity that is predictive of treatment outcome remains a further open question for cognitive hearing science to address.

## Conclusions

This investigation shows that listening difficulty has a pronounced effect on later mnemonic retention of thematically organized lists of words. This result is consistent with the view that identification of speech-in-noise adversely affects the encoding, storage, and processing of the spoken information (McCoy et al., 2005; Kjellberg et al., 2008). Further this view is consistent with noise adversely affecting semantic processes (gist-processing or relational-processing), semantic clustering, theme recall, and false recall of the critical words. All these indices of semantic processing are diminished following degraded speech presented during study. Further, the memory “gap” between intelligibility and memory is of greater magnitude than previously observed, possibly owing to the rich semantic nature of the to-be-remembered material. That gap is also greater than in previous investigations because the materials were semantically richer that the words used in those previous experiments (Kjellberg et al., 2008). Cognition, particularly in relation to semantic processes, therefore is particularly vulnerable to listening conditions during study. The results illustrate the importance of the dynamic interplay between human cognition and auditory processing. These findings are generally consistent with the assertion that adverse listening conditions recruit explicit working memory processes that, as a consequence, reduce the capacity for the efficient operation of episodic memory processes (Rönnberg et al., 2008). Finally, the results reported here are important to bear in mind when discussing acoustical norms for classrooms and other premises within which the understanding and memory for spoken information is vital: Guidelines should neither relate simply to the signal being heard, nor to the memory function for lists of unrelated words, but should also concern memory for material from which meaning is to be extracted and elaborated. The development of a hearing-cognition instrument to take into account listening and memory functions is therefore a priority area.

### Conflict of interest statement

The authors declare that the research was conducted in the absence of any commercial or financial relationships that could be construed as a potential conflict of interest.

## Footnotes

^1^ An initial pilot study was undertaken using the same methodology as outlined in the foregoing, with the exception that participants had to merely shadow (repeat back) spoken words during presentation. Eight participants (6 male; mean age 31.4 years [*SD* = 2.7]) from the University of Gävle took part in return for a cinema ticket. Each participant self-reported normal hearing and reported Swedish as a first language. Participants were randomly assigned to one of the four orders as matching that used in the study proper. These volunteers correctly repeated back 34.35 (*SE* = 0.11) words per list in the no noise condition and 32.78 (*SE* = 0.09) words per list in the noise condition. This difference was statistically significant, *t*(7) = 16.42, *p* < 0.001, *CI*_0.95_ = [1.35, 1.80]. Further analyses of the data revealed that the participants experienced difficulty only in repeating back infrequent words that were typically weak in terms of those words' backward-associative strength to the critical item. Backward associative strength is the strength of normative association from a list word (e.g., thread) to the critical word (e.g., needle) as indexed by the probability of a list word eliciting the critical word in a word association task. Backward-associative strength is the most important factor in determining false recall of the critical item (Roediger et al., 2001). Consequently, the identification of words high in backward associative strength to the critical item was unaffected by the noise. In turn, the SNR in the noise condition—which impaired identification only of weak associates of the critical lure—was not materially affected by the gist-based, or relational, processing responsible for eliciting the critical lure. Identification of the strong associates, the processing of which prime production of the critical lure went unaffected by the presence of noise. The SNR of 5 decibels was therefore deemed appropriate for use in the experiment proper.
