# Corrigendum: Failing to get the gist of what's being said: background noise impairs higher-order cognitive processing

**DOI:** 10.3389/fpsyg.2017.00390

**Published:** 2017-03-28

**Authors:** John E. Marsh, Robert Ljung, Anatole Nöstl, Emma Threadgold, Tom A. Campbell

**Affiliations:** ^1^Department of Building, Energy, and Environmental Engineering, Faculty of Engineering and Sustainable Development, University of GävleGävle, Sweden; ^2^School of Psychology, University of Central LancashirePreston, Lancashire, UK; ^3^Psychology, City UniversityLondon, UK; ^4^Neuroscience Center, University of HelsinkiHelsinki, Finland

**Keywords:** noise, elaborative processing, false recall, semantic clustering, speech intelligibility

## Error in table

In the original article, there was a mistake in Table 1 as published. Due to a tabulation error, the total number of critical lures recalled was reported incorrectly. The corrected Table 1 appears below. The authors apologize for this error and state that this does not change the scientific conclusions of the article in any way.

**Table 1:** **Mean recall performance for the four recall measures as a function of two background conditions (no noise vs. noise) used in the study**.

**Dependent measure**	**No noise**		**Noise**	
	***M***	***SD***	***M***	***SD***
Mean number of spoken words correctly recalled per list	10.45	0.67	8.78	0.58
Mean number of spoken words per theme correctly recalled per list	4.41	1.09	3.60	0.89
Mean number of themes correctly recalled per list	2.62	0.34	2.48	0.42
Total number of critical lures recalled	2.42	0.37	1.46	0.19
Thematic (Semantic)-clustering (*Z* scores)	2.43	0.20	1.95	0.16
Total number of critical lures recalled	2.42	0.37	1.46	0.19

### Conflict of interest statement

The authors declare that the research was conducted in the absence of any commercial or financial relationships that could be construed as a potential conflict of interest.

