# Supplementary material for: Failing to get the gist of what's being said: background noise impairs higher-order cognitive processing
Source: Front Psychol. 2015 May 21;6:548. doi: 10.3389/fpsyg.2015.00548 (PMC4439538; doi:10.3389/fpsyg.2015.00548)
Supplement: Supplementary file 1 [file DataSheet1.PDF]

Appendix A.

Target items

**List 1**	**List 2**	**List 3**	**List 4**	**List 5**
kort	röd	fin	jul	vatten
smal	ros	stor	vilja	ebb
flaggstång	blad	skrovlig	få	å
orm	växt	muskulös	present	nilen
stång	stjälk	sandpapper	hoppas	bäck
ståtlig	bi	rå	brunn	fors
sträcka	äng	slät	be	fisk
avstånd	träd	kraftig	födelsedag	ström
reslig	lukta	sträv	ande	älv
gänglig	tulpan	tunn	fe	ganges
basketspelare	vas	rejäl	tanke	översvämning
linjal	bukett	späd	trollspö	sjö
jobb	tråd	väg	låg	mörk
pengar	sy	stad	droger	stearin
slita	vass	kullersten	knark	lampa
fritid	stick	asfalt	torn	sol
ledig	öga	bilar	skyskrapa	blond
lön	spruta	trottoar	kulle	dag
inkomst	blod	hemma	eiffeltornet	värme
kontor	hål	rak	gräs	advent
semester	höstack	lykta	marijuana	låga
fabrik	akupunktur	aveny	påtänd	sken
dator	spetsig	nummer	stege	tändsticka
sysselsättning	häftstift	trafik	svindlande	levande
dal	bord	små	hård	godis
snö	sitta	leka	kudde	gullig
topp	dyna	vuxna	len	ful
alper	träd	dagis	nalle	socker
sten	pall	baby	filt	vacker
klättra	kök	framtid	kramdjur	sur
höjd	möbel	mamma	bomull	snygg
klippa	soffa	familj	päls	kola
skidor	bar	syskon	gosig	salt
fjäll	bekväm	joller	ull	choklad
norge	bänk	napp	moln	smak
granit	fåtölj	vagn	fluffig	rar

**List 6**	**List 7**	**List 8**	**List 9**	**List 10**
spik	mat	sommar	golv	skrämd
verktyg	smör	vingar	frans	ensam
slå	äta	puppa	rya	modig
tor	skiva	larv	persisk	skraj
snickardon	frukost	fladdrar	dammsuga	feg
skruvmejsel	limpa	mask	trasa	trygg
tumme	macka	skör	randig	ledsen
såg	kniv	vår	hall	ängslig
slöjd	rosta	håv	äkta	osäker

stål	baka	kokong	tvätta	darra
städ	smulor	mygga	orientalisk	orolig
öra	torrt	silke	ombonat	obehagligt
rund	säng	lätt	pojke	glas
månen	dröm	bly	flätor	utsikt
planet	sova	tjock	tös	hus
klot	trött	vikt	ung	gardin
tellus	vaken	kilo	tjej	ruta
universum	vila	elefant	kjol	dörr
världen	snarka	fet	klänning	karm
glob	pigg	hantel	oskuld	putsa
himlen	slumra	bak	hopprep	genomskinligt
brun	gäspa	järn	dockor	luft
rymden	dvala	massiv	dotter	öppet
miljö	tupplur	bastant	fräknar	spröjs
vit	tå	toner	fyrkant	snabb
natt	hand	noter	matte	snigel
katt	skor	gitarr	cirkel	slö
färg	kroppsdel	sång	geometri	trög
sammet	nagel	instrument	kub	seg
dyster	svett	mozart	figur	sakta
sorg	vårta	dans	rot	tråkig
hatt	strumpa	radio	triangel	sköldpadda
hår	ankel	lyssna	rektangel	fort
kol	häl	ljuv	form	lat
begravning	boll	klassisk	låda	sölig
död	sula	piano	meter	promenad

Critical Items

Swedish

**List 1**	**List 2**	**List 3**	**List 4**	**List 5**
Lång	Blomma	Grov	Önska	Flod
Arbete	Nål	Gata	Hög	Ljus
Berg	Stol	Barn	Mjuk	Söt
**List 6**	**List 7**	**List 8**	**List 9**	**List 10**
Hammare	Bröd	Fjäril	Matta	Rädd
Jorden	Sömn	Tung	Flicka	Fönster
Svart	Fot	Musik	Kvadrat	Långsam

English Translation

**List 1**	**List 2**	**List 3**	**List 4**	**List 5**
Tall	Flower	Thick	Wish	River
Work	Needle	Street	High	Light
Mountain	Chair	Child	Soft	Sweet
**List 6**	**List 7**	**List 8**	**List 9**	**List 10**
Hammer	Bread	Butterfly	Carpet	Frightened
Earth	Sleep	Heavy	Girl	Window
Black	Foot	Music	Quadrant	Slow
